# A Comparative Analysis of CSF and the Blood Levels of Monoamines As Neurohormones in Rats during Ontogenesis

**DOI:** 10.32607/actanaturae.11516

**Published:** 2021

**Authors:** A. R. Murtazina, N. S. Bondarenko, T. S. Pronina, K. I. Chandran, V. V. Bogdanov, L. K. Dilmukhametova, M. V. Ugrumov

**Affiliations:** Institute of Developmental Biology RAS, Moscow, 119334 Russia

**Keywords:** rat, brain, cerebrospinal fluid, plasma, monoamines, ontogenesis

## Abstract

According to the literature, the cerebrospinal fluid (CSF) in the cerebral ventricles contains numerous neuron-derived physiologically active substances that can function as neurohormones and contribute to volume neurotransmission in the periventricular region of the brain. This study was aimed at carrying out a comparative analysis of CSF and the blood levels of monoamines in rats during ontogenesis as an indicator of age-related characteristics of monoamine transport to body fluids and their function as neurohormones in volume neurotransmission in the periventricular region of the brain. We have shown that CSF in the perinatal period and adulthood contains the most functionally significant monoamines: dopamine, noradrenaline, and serotonin. A comparison of the monoamine levels in the CSF and blood of animals of different age groups revealed that CSF contains monoamines of predominantly neuronal (cerebral) origin and almost no monoamines derived from the general circulation. We also established that monoamines are found in the CSF at physiologically active levels that allow them to act as neurohormones in both reversible volume neurotransmission in the adult brain and irreversible regulation of brain development in the perinatal period.

## INTRODUCTION

Monoamine dopamine (DA), noradrenaline (NA), and serotonin (5-hydroxytryptamine, 5-HT), which are synthesized in brain neurons, play an important role in the regulation of brain function via volume neurotransmission (action on the entire neuronal surface) and synaptic neurotransmission (action in the synaptic area) [1]. In adult animals, brain monoamines are responsible for the reversible autoregulation of neurons synthesizing them and the regulation of other "-ergic" neurons. In the perinatal period of ontogenesis, monoamines act on the same receptors on target neurons and have an irreversible morphogenetic effect on the development of these neurons and the brain as a whole [2, 3, 4, 5].

There is evidence that neuron-derived monoamines in the cerebrospinal fluid (CSF) in the cerebral ventricles enter the brain and participate as neurohormones in volume neurotransmission due to the absence of a CSF–brain barrier for them [1, 6]. Although monoamines are also synthesized in peripheral organs and reach the blood vessels, their neurohormonal effect on the brain can take place only before closure of the blood–brain barrier, which occurs in the early postnatal period [7]. However, an insignificant exchange of monoamines between the CSF and blood is possible in ontogenesis: (a) in the area of the choroid plexuses in the lateral ventricles, where substances enter the CSF from the blood; (b) at the border between ventricles in the caudal region of the brain and the vascular system; (c) in the circumventricular organs of the brain lacking the blood–brain barrier [7, 8].

Despite the abundance of evidence to the presence of physiologically active substances, including monoamines, in the CSF and blood, the pattern of changes in the monoamine level in these body fluids during ontogenesis has not been elucidated yet. In addition, the monoamine level gradient at the CSF–blood border during various stages of ontogenesis has never been assessed before. Considering our recent data on the absence of a CSF barrier for monoamines during ontogenesis in rats [9], the levels of monoamines should be the same in the intercellular space in the periventricular region of the brain and in the CSF. Finding an answer to these questions will make it possible to determine at what stages of ontogenesis the CSF level of monoamines is high enough for them to act as neurohormones in the regulation of brain development and function.

Based on the above, the purpose of our study was to perform a comparative analysis of the CSF and blood levels of monoamines in rats during ontogenesis as an indicator of age-related characteristics of monoamine transport to body fluids and their participation as neurohormones in volume neurotransmission in the brain. To achieve this goal, the following tasks were set: (a) determine the level of monoamines DA, NA, adrenaline (A), and 5-HT as an indicator of the secretory activity of the corresponding neurons in rat CSF on embryonic day 18 (E18) and postnatal days 5 (P5) and 30 (P30); (b) determine the plasma levels of monoamines in these animals; (c) evaluate the ratio of CSF to the plasma levels of the monoamines as an integrated index of the existence of barriers for monoamines between the cerebral ventricles and the general circulation.

## EXPERIMENTAL PROCEDURES

**Animals**

**Fig. 1:**
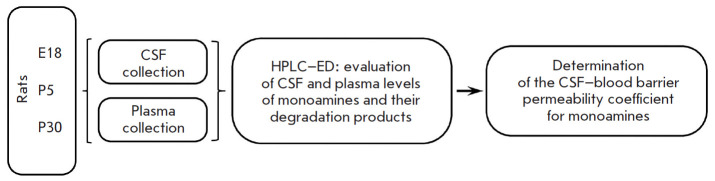
Scheme of experiments in rats on embryonic day 18 (E18) and postnatal days 5 (P5) and 30 (P30): CSF and plasma collection, evaluation of the CSF and plasma levels of monoamines and their degradation products, determination of the CSF–blood barrier permeability coefficient for monoamines. HPLC–ED – high-performance liquid chromatography with electrochemical detection

The study was performed in female and male Wistar rats on E18 and male Wistar rats on P5 and P30 (Fig. 1). To obtain dated offspring, pregnant female rats weighing 250–350 g were used. The day when sperm was detected in the vaginal smear was considered E1; the day of pup birth was considered P1. The animals were maintained under standard vivarium conditions with a 12-h light/dark cycle and free access to food and water. The experiments were carried out in accordance with the guidelines of the National Institutes of Health (NIH Guide for the Care and Use of Laboratory Animals) and the Bioethics Committee of the Koltsov Institute of Developmental Biology (Minutes No. 3 dated September 10, 2020, and Minutes No. 44 dated December 24, 2020).

All animal procedures were performed under anesthesia with either chloral hydrate (Sigma, USA) at a dose of either 100 mg/kg on P5 and 400 mg/kg on P30 and E18 or 1% isoflurane on P30 (Laboratorios Karizoo, Spain).

**Collection of rat CSF and blood on E18, P5, and P30**

CFS was collected from rats on E18 (n = 112), P5 (n = 30), and P30 (n = 20) (Fig. 1). On gestation day 18, the rats were subjected to laparotomy, fetuses were removed from the uterus leaving the umbilical cord intact. After that, a glass micropipette connected by a Teflon tube to a Hamilton syringe filled with a saline solution was inserted into each of the fetal lateral ventricles according to [10]. The micropipette’s tip was filled with a small air bubble to avoid saline mixing with the CSF. An average of 1.5 ± 0.5 μL of CSF was obtained from both fetal ventricles.

**Fig. 2:**
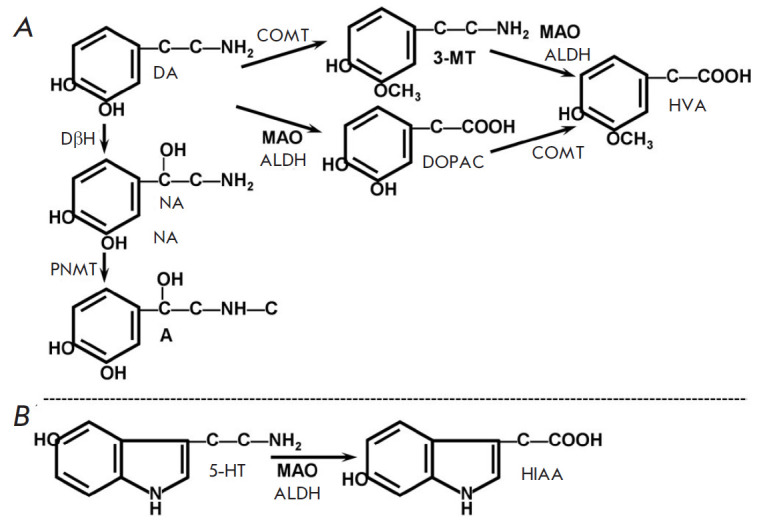
Monoamines and some products of their degradation. DA – dopamine; NA – noradrenaline; A – adrenaline; DOPAC – 3,4-dihydroxyphenylacetic acid; 3-MT – 3-methoxytyramine; HVA – homovanillic acid; 5-HT – serotonin; HIAA – 5-hydroxyindoleacetic acid

CSF was collected from the cisterna magna of rats on P5 and P30 according to the previously described technique [11]. For this, an animal’s head was secured in a stereotaxic apparatus (Narishige Scientific Instrument Lab., Japan) to access the cisterna magna. Then, a glass micropipette connected to a Hamilton syringe was inserted stereotaxically into the cisterna magna using the system described above. A total of 25 ± 10 and 55 ± 15 μl of CSF were collected from each rat on P5 and P30, respectively. After that, HClO_4_ and 3,4-dihydroxybenzylamine (DHBA) (Sigma, USA), which is an internal standard for the determination of monoamines and their degradation products, were added to the CSF samples to final concentrations of 0.1 M and 25 pM, respectively. CSF obtained from 14 fetuses was used as the E18 sample, and CSF of three animals was used as P5 and P30 samples. CSF samples were frozen in liquid nitrogen and stored at –70°C prior to determination of monoamines DA, NA, and 5-HT and their main degradation products 3,4-dihydroxyphenylacetic acid (DOPAC), 3-methoxytyramine (3-MT), homovanillic acid (HVA), and 5-hydroxyindoleacetic acid (HIAA) (Fig. 2).

On P30, CSF was sampled from both the cisterna magna and lateral ventricles. For this, a guide cannula for a microdialysis probe (CMA-11 Guide Cannula, CMA, Sweden) was inserted stereotaxically into the lateral ventricle of the brain in rats (n = 4) anesthetized with isoflurane based on coordinates calculated according to the rat brain atlas (–0.4 mm caudal and 1.4 mm lateral to the bregma; 2.2 mm deep) [12]. The cannula was fixed to the skull bone using micro bone screws and dental cement (Protakril-M, Ukraine). After 48 h, a microdialysis probe (CMA 11 55 kDa Microdialysis Probe, CMA, Sweden) filled with artificial CSF (147 mM NaCl, 2.7 mM KCl, 1 mM MgCl_2_, and 1.2 mM CaCl_2_) was inserted into the guide cannula. The probe was connected to a CMA 4004 Microdialysis Pump (CMA, Sweden) through Teflon tubes. Microdialysis was initiated 3 h after probe insertion: lateral ventricles were perfused for 20 min at a flow rate of 2 μl/min. The resulting dialysis sample was mixed with 4 μl of 1 N HClO_4_, frozen in liquid nitrogen and stored at –70°C until determination of monoamines and their degradation products (Fig. 2).

After CSF was sampled, blood was collected from the left ventricle of the heart of the same animals using an insulin syringe under chloral hydrate anesthesia. The volume of blood samples collected from the animals on days E18, P5, and P30 was 30 ± 5, 100 ± 10, and 2,000 ± 20 μL, respectively. Blood samples were supplemented with 5% ethylenediaminetetraacetic acid (EDTA) (Sigma, USA) and 10% sodium metabisulfite (Sigma). Samples were centrifuged at 1,350 g and 4°C for 10 min, the supernatant (plasma) was collected and mixed with 10% 1 N HClO_4_ and 25 pmol of DHBA. Plasma was centrifuged at 16,500 g and 4°C for 20 min, the supernatant was frozen in liquid nitrogen and stored at –70°C until determination of monoamines and their degradation products (Fig. 2).

**High-performance liquid chromatography with electrochemical detection**

The concentration of monoamines and their degradation products in CSF, plasma, and microdialysis samples was determined using high-performance liquid chromatography with electrochemical detection. CSF and plasma samples were divided into two parts. One part was extracted by precipitating catecholamines and their degradation products onto aluminum oxide, another part was not precipitated but was used directly to measure the 5-HT and HIAA levels. Microdialysis samples were not precipitated.

The test substances were separated using a 4 × 100-mm reversed-phase ReproSil-Pur ODS-3 column with a pore diameter of 3 μm (Dr. Majsch GMBH, Germany) at 28°C and a mobile phase flow rate of 1 ml/min using a LC-20ADsp Liquid Chromatograph Pump (Shimadzu, Japan) at a potential of 850 mV. Citrate-phosphate buffer (0.1 M; pH 2.58) containing 0.3 mM sodium octanesulfonate, 0.1 mM EDTA, and 8% acetonitrile (all Sigma reagents) was used as the mobile phase. Monoamines were determined using a DECADE II electrochemical detector (AntecLeyden, Netherlands) with a glassy carbon working electrode (0.85 V) and a silver chloride reference electrode. The peaks of monoamines and their metabolites were determined based on their retention time in a standard solution.

**Statistical data analysis**

A statistical analysis was performed using the GraphPad Prism 6.0 software (USA). Data are presented as the mean ± standard error of the mean (mean ± SEM). Differences were considered statistically significant at p < 0.05; 0.05 < p < 0.1 was regarded as a tendency to differences; the differences were considered insignificant at p > 0.1. The statistical significance of the results was determined using the parametric Student’s t-test (t-test) and the nonparametric Mann–Whitney U-test (U-test); the Bonferroni correction was used for multiple testing.

## RESULTS

**CSF levels of catecholamines, 5-HT, and their degradation products in the cisterna magna in rats during ontogenesis**

**Fig. 3:**
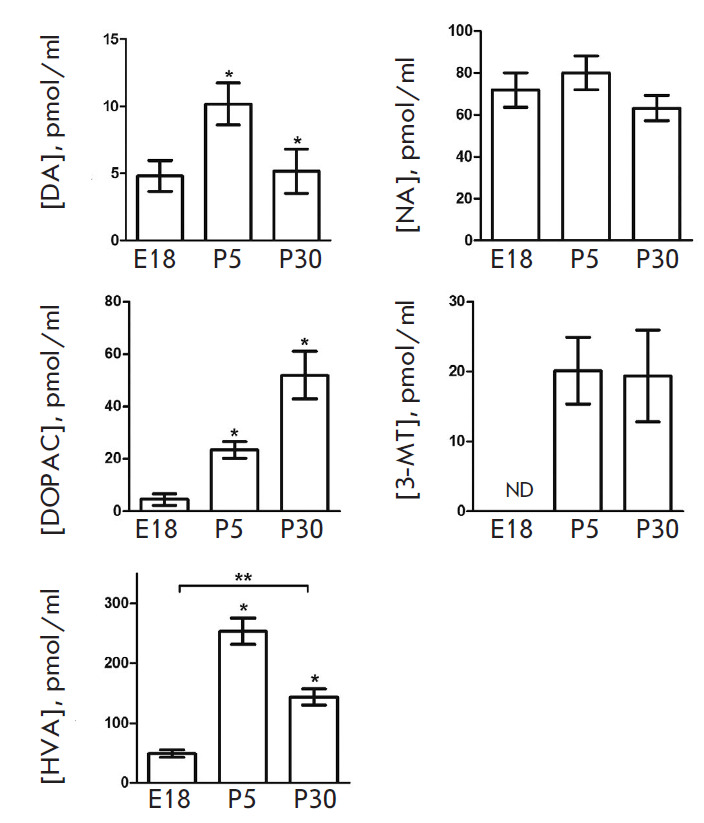
CSF levels of catecholamines and some products of their degradation in rats on embryonic day 18 (E18) and postnatal days 5 (P5) and 30 (P30). DA – dopamine; NA – noradrenaline; DOPAC – 3,4-dihydroxyphenylacetic acid; 3-MT – 3-methoxytyramine; HVA – homovanillic acid. *p < 0.05 – comparison with the previous age; **p < 0.05 – comparison of the selected parameters; ND – not detected

The DA level is approximately 5 pmol/ml on E18; it increases twofold on P5 and reaches that of fetuses on P30 (Fig. 3).

The NA level does not change during the entire period of ontogenesis and remains at a high level (~70 pmol/ml) in all age groups. In contrast to DA and NA, A was detected in the CSF during neither the prenatal nor postnatal period.

In addition to catecholamines, their degradation products 3-MT (except for day E18), DOPAC, and HVA are found in the CSF of rats of all age groups (Fig. 3). Although 3-MT is not detected in the fetuses, it is observed at a fairly high level (~20 pmol/ml) in the CSF on P5 and P30. In contrast to 3-MT, DOPAC is detected as early as E18 at a concentration of almost 4.5 pmol/ml. This parameter increases about fivefold on P5 and decreases by 55% on P30. The end product of DA degradation, HVA, is detected at a high level (almost 50 pmol/ml) in the CSF as early as E18. This parameter increases about fivefold on P5 and decreases by 45% on P30.

**Fig. 4:**
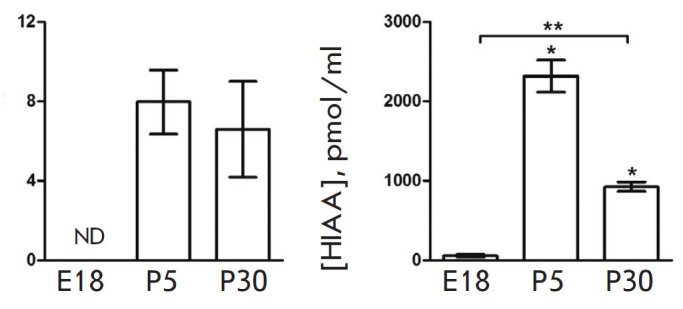
CSF levels of serotonin (5-HT) and its degradation product 5-hydroxyindoleacetic acid (HIAA) in rats on embryonic day 18 (E18) and postnatal days 5 (P5) and 30 (P30). *p  < 0.05 – comparison with the previous age; **p  < 0.05 – comparison of the selected parameters; ND – not detected

No 5-HT is detected in rat CSF on E18; however, a high CSF level of 5-HT (8 pmol/ml) is noted on P5 and P30 (Fig. 4). The level of the end product of 5-HT degradation, HIAA, is quite high on E18; it exceeds that of 5-HT by almost 300 times. On P30, the CSF level of HIAA is reduced by more than twofold compared to P5 (Fig. 4).

**Monoamines and their degradation products in the lateral ventricles of rats on P30**

Of all monoamines and their degradation products, only DOPAC and HIAA were detected by microdialysis in the lateral ventricles of the rat brain on P30. At least three measurements were made to establish the baseline level of the test substances. The following DOPAC and HIAA levels were detected in dialysis samples: 5.2 ± 1.1 and 105.7 ± 14.8 pmol/ml, respectively.

**Levels of catecholamines, 5-HT, and their degradation products in rat plasma during ontogenesis**

**Fig. 5:**
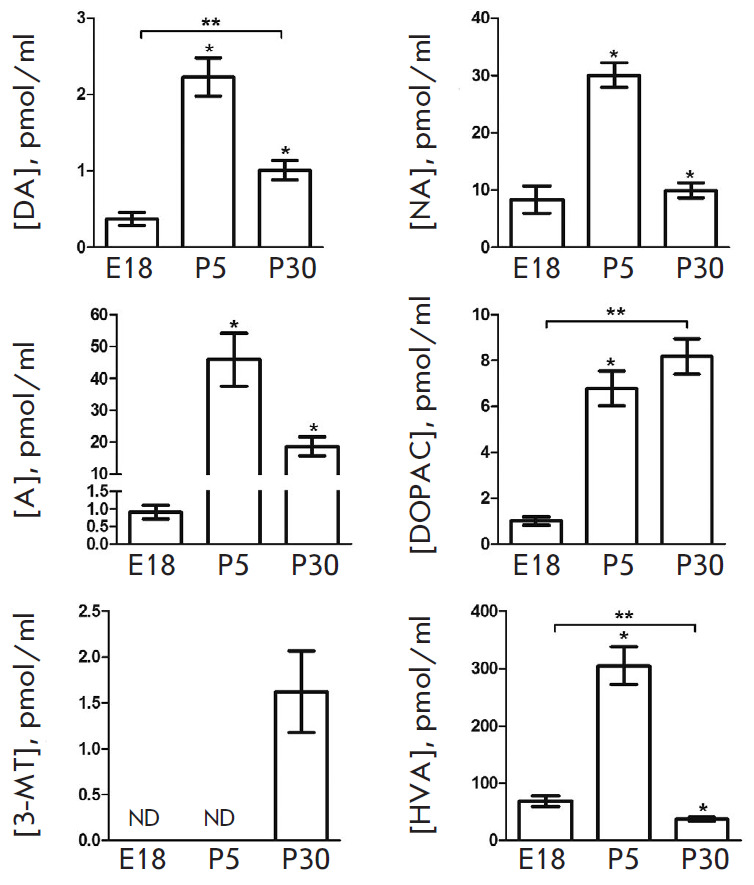
Plasma levels of catecholamines and some products of their degradation in rats on embryonic day 18 (E18) and postnatal days 5 (P5) and 30 (P30). DA – dopamine; NA – noradrenaline; A – adrenaline; DOPAC – 3,4-dihydroxyphenylacetic acid; 3-MT – 3-methoxytyramine; HVA – homovanillic acid. *p  < 0.05 – comparison with the previous age; **p  < 0.05 – comparison of the selected parameters; ND – not detected

The plasma level of DA in rats on E18 does not exceed 0.4 pmol/ml, it increases sixfold on P5, and decreases about twofold on P30 (Fig. 5). The pattern of A level changes during ontogenesis is about the same as that of DA: it increases 3.5-fold from E18 to P5 and then decreases threefold on P30. The NA level is an order of magnitude higher than that of DA in all age groups. Unlike for the CSF, A is determined in the plasma: its level increases by more than 50-fold from E18 to P5 and decreases 2.5-fold by P30. Both catecholamines and their degradation products are found in the plasma. While 3-MT is detected in the plasma only on P30, DOPAC (at a low concentration) is observed as early as E18. The DOPAC level increases significantly by P5 and remains the same by P30. The level of HVA, the end product of DA degradation, increases more than fourfold from E18 to P5 and becomes lower than that of the fetuses by P30 (Fig. 5).

**Fig. 6:**
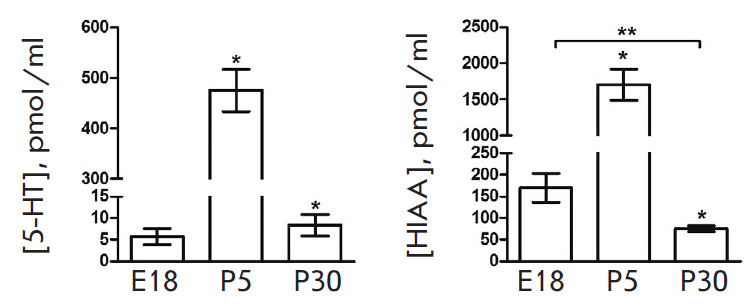
Plasma levels of serotonin (5-HT) and its degradation product 5-hydroxyindoleacetic acid (HIAA) in rats on embryonic day 18 (E18) and postnatal days 5 (P5) and 30 (P30). *p  < 0.05 – comparison with the previous age; **p  < 0.05 – comparison of the selected parameters

The plasma level of 5-HT is approximately 5.5 pmol/ml on E18 (Fig. 6). It increases 85-fold by P5 and then decreases almost to that of the embryos on P30. The HIAA level changes in a similar way: it increases 10-fold from E18 to P5 and becomes two times lower than that during the embryonic period on P30.

**Ratio of CSF to the plasma levels of monoamines and their degradation products in rats during ontogenesis**

**Fig. 7:**
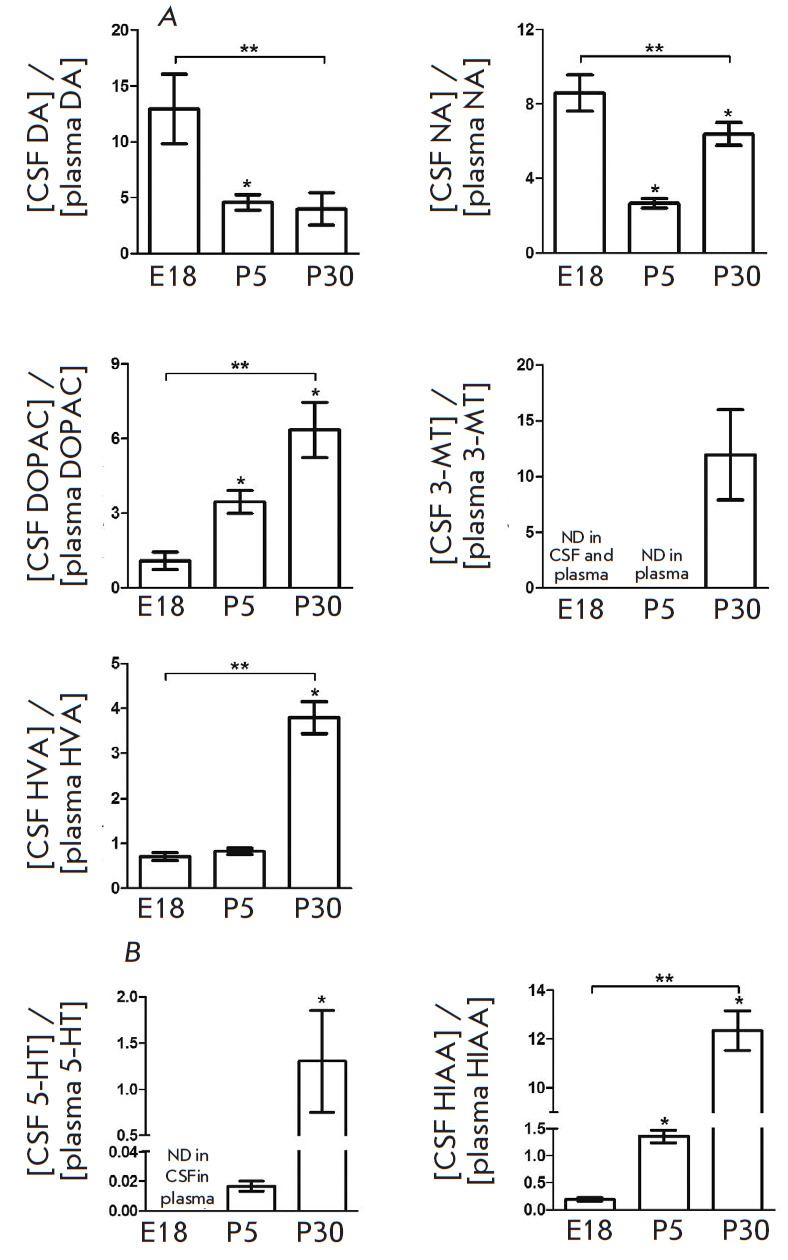
Ratio of CSF to the plasma levels of catecholamines (*A*), 5-HT (*B*), and some products of their degradation (*A, B*) in rats on embryonic day 18 (E18) and postnatal days 5 (P5) and 30 (P30). DOPA – 3,4-dihydroxyphenylacetic acid; 3-MT – 3-methoxytyramine; HVA – homovanillic acid; 5-HIAA – 5-hydroxyindoleacetic acid. *p  < 0.05 – comparison with the previous age; **p  < 0.05 – comparison of the selected parameters; ND – not detected

The CSF levels of DA, NA, and their degradation products are many times higher than their plasma levels in all age groups (Fig. 7A). However, maximum differences are noted during different periods of ontogenesis. For instance, the peaks in DA and NA levels are observed on E18, while DOPAC, 3-MT, and HVA levels reach their maximum on P30.

The CSF levels of 5-HT and HIAA exceed those in plasma on P30 (for 5-HT), P5 and P30 (HIAA) (Fig. 7B). It should be noted that the CSF level of 5-HT on P5 is 60 times lower than that in plasma.

## DISCUSSION

The main goal of this work was to perform a comparative analysis of the CSF and blood levels of monoamines in rats during ontogenesis as an indicator of age-related characteristics of monoamine release into the body fluids and their role as neurohormones in brain development and function. Special attention is paid to the CSF as a body fluid that, on the one hand, receives monoamines from the brain and, on the other hand, releases monoamines to the periventricular region of the brain in the absence of a CSF–brain barrier, which then acts as neurohormones in volume neurotransmission. The discovery of the CSF-contacting neurons was the historical basis for hypothesizing the release of physiologically active substances from the brain to the CSF [8, 13].

**CSF monoamines acting as neurohormones are potential participants in volume neurotransmission and the morphogenetic control of brain development**

Despite the fact that dozens of neurotransmitters and neuromodulators are synthesized in brain neurons, monoamines DA, NA, and A are the most common classical neurotransmitters in the brain. These monoamines are involved in the regulation of the functional activity of target neurons in adult animals and the regulation of neuron and brain development in the perinatal period [2, 3, 4, 5].

Based on the concept of qualitative differences in monoamine action at different stages of ontogenesis, the CSF level of monoamines was determined in rats at three age periods. The first age group included E18 rats, since the following events take place by this time: (a) formation of neurons from progenitor cells is completed; (b) the differentiating neurons migrate to the sites of their final localization in the brain; (c) neurons express a specific phenotype; and (d) axons of the differentiating neurons reach brain ventricles and blood vessels in the circumventricular organs, forming pathways for neurohormones to enter the CSF (axo-ventricular contacts) and blood vessels (axo-vascular contacts). The second age group included P5 rats. The following events take place by this time: (a) migration of differentiating neurons to the sites of their final localization is completed; (b) axo-ventricular and axo-vascular contacts are established; and (c) afferent synaptic innervation of neurons continues to form. The third age group included P30 rats. The following events take place by this time: (a) formation of synaptic contacts ends; (b) formation of the blood–brain barrier, which prevents penetration of most non-lipid neurotransmitters from the brain into the blood and vice versa, is completed [14, 15, 16, 17].

From the standpoint of the neurohormonal effect of CSF monoamines on the brain neurons, the CSF level of monoamines is considered the most important functional parameter. Indeed, the previous, mainly in vitro, studies demonstrated that monoamines have a neurotransmitter effect on neurons at a wide range of concentrations: from 10-11 to 10-8 M [18]. Moreover, monoamines can affect neurons at even lower concentrations in vivo [19, 20, 21].

At the first stage of our study, it was necessary to determine whether the qualitative composition and level of monoamines change with the CSF flow from the lateral ventricles, where CSF is formed as a result of plasma filtration from the vessels of the choroid plexus to the cisterna magna: the caudal part of the ventricular system. For this, we compared the composition of monoamines and their degradation products in rat CSF obtained from the lateral ventricles by microdialysis and rat CSF of the cisterna magna collected on P30 using a micropipette. We found all monoamines and their degradation products detected by us in the cisterna magna, while only some products of monoamine degradation were observed in the lateral ventricles: DOPAC and HIAA. These data indicate that monoamines enter the CSF not from the plasma of the choroid plexus vessels and the nerve tissue surrounding the lateral ventricles but mainly from the nervous tissue caudal to the lateral ventricles of the brain.

Further, the composition of CSF obtained from the cisterna magna only was determined. Catecholamines DA and NA were found in the CSF of rats of all age groups; however, changes in their levels were significant with age. For instance, NA is present in the CSF at a significant level (7.2 × 10^-8^ M) on E18; it remains the same on P5 (6.3 × 10^-8^ M) and P30 (6.3 × 10^-8^ M). The obtained data indicate that noradrenergic neurons secrete monoamines into the CSF during the prenatal and postnatal periods. This assumption is supported by the fact that, according to the in vitro data, NA at approximately the same level as that of CSF can exert a neurotransmitter effect on neurons in adult rats [22, 23, 24]. Considering also the fact that receptors for NA and other monoamines are expressed even during the prenatal period [25, 26, 27], our data suggest that CSF NA is not only able to participate as a neurohormone in volume neurotransmission in adult animals but also exert a morphogenetic effect on the brain neurons in the perinatal period.

Age-related changes in the CSF levels of DA and NA have fundamental differences. The first difference is that the CSF level of DA (0.3 × 10-9 M) is at least an order of magnitude lower than that of NA (0.48 × 10-8 M) on E18. However, this does not reduce the possible morphogenetic effect of DA on target neurons. The CSF level of DA increases twofold (1 × 10-8 M) by P5, although it remains significantly lower than that of NA. It should be noted that the level of DA could have increased in the perinatal period (E18–P5) much more, if the activity of DA breakdown enzymes, MAO and COMT, had not increased simultaneously. This is evidenced by a significant increase in the levels of the products of MAO and COMT enzymatic activity: DOPA, 3-MT, and HVA in the perinatal period. Nevertheless, an increase in the CSF level of DA on P5 significantly increases the probability of its morphogenetic effect on differentiating target neurons and brain development in general [28]. The second difference in age-related changes between the CSF levels of DA and NA is a twofold decrease in the DA level on P30 compared to P5. This is an important, albeit indirect, indicator that the CSF DA can have a morphogenetic effect on target neurons, mainly in the early postnatal period.

The age-related pattern of the CSF level of 5-HT differs significantly from that of catecholamines. For instance, almost no 5-HT is detected in the CSF on E18, while its level almost reaches that of DA on P5. The CSF level of 5-HT could have increased to an even greater extent on P5, if not for a significant increase in the activity of the 5-HT degradation enzyme MAO by that period, as indicated by the high level of the product of its enzymatic breakdown: HIAA. The 5-HT level remains the same by P30. The above data indicates that the CSF 5-HT can both participate in volume neurotransmission in the postnatal period and regulate the development of target neurons and the brain in general in the early postnatal period in rats.

**The brain is the only source of CSF monoamines during ontogenesis**

As shown in our study, the CSF of rats on P5 and P30 contains monoamines at physiologically active levels. Furthermore, in contrast to 5-HT, catecholamines are also present in the CSF on E18. However, these data cannot serve as direct evidence that the brain is the only source of CSF monoamines. Indeed, we cannot exclude that monoamines enter the CSF not only from the brain neurons but from the bloodstream as well in the absence of a blood–brain barrier for monoamines in the perinatal period [14, 29, 30], with also taking into account the possibility of a metabolism between the CSF and blood in the choroid plexus in the lateral ventricles, in circumventricular organs, and the area of the caudal venous sinus even in adult animals [7]. In addition, one should not forget the fact that there are such important sources of monoamines as the adrenal glands, gastrointestinal tract, and the peripheral sympathetic nervous system [31, 32, 33].

An answer to the question of whether the brain is the only source of monoamines in the CSF can be found in a first approximation by calculating the integrated index of permeability of all possible barriers on the way of monoamines from the blood to the CSF in the form of the ratio of CSF to blood levels of monoamines. Three options can be considered: (1) the permeability coefficient equals to unity, indicating the absence of a barrier between the CSF and the blood; (2) the permeability coefficient is greater than unity, which indicates the presence of a barrier for monoamine entry from the CSF to the blood; (3) the permeability coefficient is less than unity, which indicates the existence of a barrier preventing monoamine entry from the blood to the CSF.

To calculate the coefficient of permeability of barriers between the CSF and blood for monoamines, both the CSF and blood levels of monoamines were measured in rats during ontogenesis. Age-related changes in the plasma levels of the monoamines DA, NA, and 5-HT were found to be similar. These monoamines were detected in the blood at insignificant levels on E18, their levels increased significantly by P5 and then decreased almost to the E18 level by P30. However, the levels of individual monoamines in each age group varied significantly. For instance, on P5, the plasma level of NA was 15 times higher than that of DA and more than 150 times lower than that of 5-HT.

Determination of the ratio of CSF to the blood levels of monoamines showed that the permeability coefficient for barriers between the CSF and blood on E18 is 8.5 and 13 for NA and DA, respectively. This means that CSF catecholamines originate from the brain. These calculations could not be carried out for 5-HT, since 5-HT is not detected in the CSF of the fetus at such a time point. Although the permeability coefficient for catecholamines is reduced significantly, it remains above unity in the postnatal period. This indicates that, during this period, catecholamines enter the CSF only from the brain neurons.

In contrast to catecholamines, the permeability coefficient for 5-HT on P5 is only slightly less than unity and, then, sharply increases by P30. This indicates that the barrier preventing the exchange of monoamines between the CSF and blood also exists for 5-HT. The most important evidence that monoamines can penetrate the barriers on their way from the blood to the CSF is the high blood level of E in rats on P5 and P30 and its absence in the CSF in rats of the same age. The obtained data confirm the existence of barriers preventing the entry of substances from the CSF into the blood and vice versa in the pre- and postnatal period [34, 35, 36].

## CONCLUSION

The following conclusions were made: (1) the cerebrospinal fluid of rats in the perinatal period and adulthood contains the most functionally significant monoamines: dopamine, noradrenaline, and serotonin; (2) the cerebrospinal fluid contains monoamines of predominantly neuronal (cerebral) origin and almost no monoamines derived from the general circulation; (3) monoamines are found in the cerebrospinal fluid at physiologically active levels that allow them to act as neurohormones in volume neurotransmission: as morphogenetic factors in irreversible regulation of neuronal development in the perinatal period and reversible regulation of the functional activity of target neurons in adult animals.

## Abbreviations

3-MT: 3-methoxytyramine
5-HT: 5-hydroxytryptamine (serotonin)
A: adrenaline
ALDH: aldehyde dehydrogenase
HVA: homovanillic acid
HIAA: 5-hydroxyindoleacetic acid
DβH: dopamine β-hydroxylase
DA: dopamine
DHBA: 3,4-dihydroxybenzylamine
DOPAC: 3,4-dihydroxyphenylacetic acid
COMT: catechol-O-methyltransferase
MAO: monoamine oxidase
NA: noradrenaline
P: postnatal day
PNMT: phenylethanolamine-N-methyltransferase
E: embryonic day
